# A direct and non-invasive method for kidney delivery of therapeutics in mice

**DOI:** 10.1016/j.mex.2018.10.019

**Published:** 2018-10-23

**Authors:** Nimshitha Pavathuparambil Abdul Manaph, Mohammed Al-Hawwas, Xin-Fu Zhou

**Affiliations:** a School of Pharmacy and Medical Sciences, Sansom Institute, University of South Australia, Adelaide, 5000, Australia; b Central Northern Adelaide Renal and Transplantation Service, Royal Adelaide Hospital, Adelaide, 5000, Australia; c School of Medicine, Faculty of Health Sciences, University of Adelaide, Adelaide, 5000, Australia

**Keywords:** Direction injection into mice kidney, Kidney, Drugs, Delivery, Surgery, Injection

## Abstract

Kidney is a vital organ that maintains the homeostasis in terms of acid-balance, toxin filtration and blood pressure control. Kidney malfunction can be fatal and the renal research administers testing pharmaceutical agents or stem cells in rodents to study their therapeutic efficacy. However, targeted delivery of agents into mice kidney is strenuous and may require laparotomy. Here we present a direct delivery method for cell transplantation or drug injection into the mice kidney. The method is simple and can be performed non-invasively with avoidance of surgical intervention on the animals. Nevertheless, this method serves as an efficient method for *in vivo* drug delivery or engraftment studies for renal research.

• Direct delivery into the kidney.
• Non-invasive method.

## Graphical abstract

**Figure:**
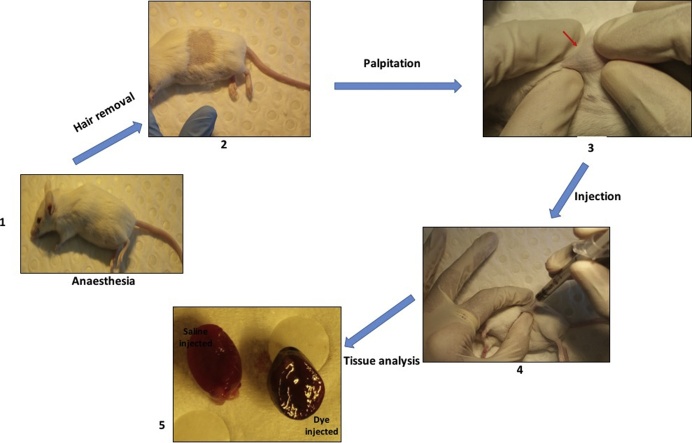

**Specifications Table**

**Table:** 

Subject area	*Select one of the following subject areas:* • Veterinary Science and Veterinary Medicine
More specific subject area	*Veterinary medicine*
Method name	*Direction injection into mice kidney*
Name and reference of original method	*Yoneko M, Kamei J, Ito CF, Kojima J. New approach for chronic renal failure model by direct kidney injection of doxorubicin in rats. Methods Find Exp Clin Pharmacol.2007; 29:6.*
Resource availability	*Critical components are indicated in the material section*

## Introduction

Drug testing is carried out in animals by intravenous, peritoneal or subcutaneous injections [1]. For nephrotic syndrome model studies, high drug concentrations are required inside the kidney [2]. In such cases, high concentration of intravenous and peritoneal drugs injection may not necessarily bring concentration of drugs high enough in the organ [3]. Surgical method of transplantation by laparotomy is an effective method for delivery into renal tissues. However, for surgically based method researchers need to cut and open the abdomen to allow precise injection into the organ.

In the past few years, stem cells therapy has been suggested to treat diabetic patients. For diabetic studies using stem cells, the cells are transplanted inside the kidney capsule, generally *via* surgical intervention. This has been considered as a standard procedure that allows long-term survival of cells inside the kidney capsule. Firstly, the abdomen is exposed by laparotomy and the kidney is taken out carefully. Following that a small incision is made through the kidney membrane and the cells are then transfused into the renal capsule [4]. However, the main downside of this strategy is the high impact on animal welfare. Also, typically for human stem cell research, immunocompromised mice are used and consequently, the animals are deteriorated. In such cases, performing surgery can considerably compromise the survival of the animals [5]. Surgically based delivery method also increases the chance of wound infection from stitches and the need for analgesics. Furthermore, the surgically based method is not convenient for studies where more than one-shot delivery is required. Therefore, kidney-targeted delivery is of great significance and efficient delivery strategy needs to be implemented for research. In this study, we demonstrate a modified method of injection that allows precise and reliable delivery of therapeutics and cells straight into the kidney without surgery.

## Material and methods

### Animals

All procedures were pre-approved by the animal ethics committee of University of

South Australia (ethics no: U04-15). The strains of the animals used for the study were C57BL/6 and NOD/SCID.

### Materials

The materials required for the injection were a heat pad, injectable anaesthetic agent (ketamine-10 mg/kg), cotton gauze, 70% alcohol, 0.5 ml insulin syringe. The cells for injection were pre-tagged with green fluorescent protein (GFP).

### Procedure

**Fig. 1:**
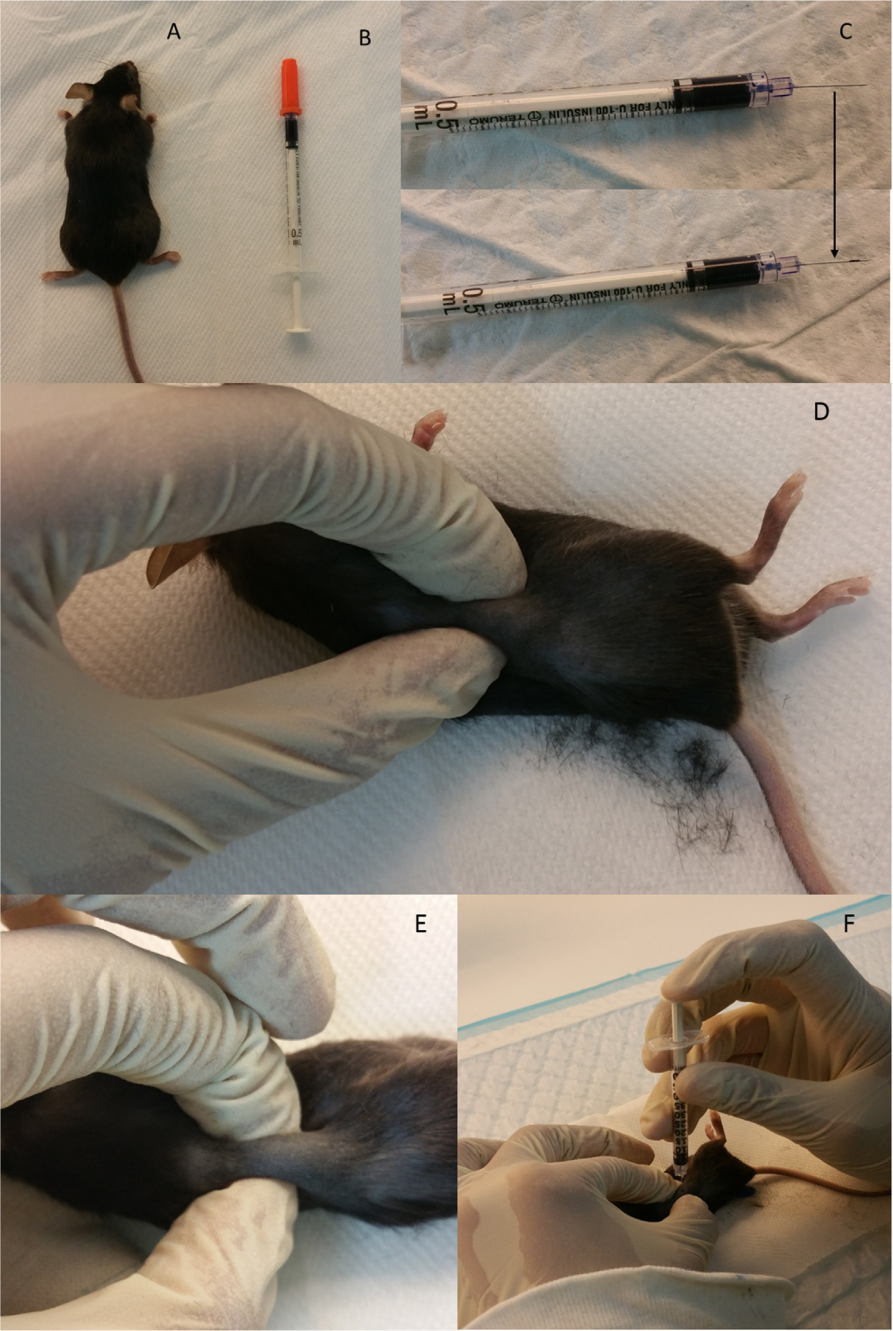
**Key steps in the procedure as demonstrated on C57 mice. A:** Mice anesthetised by subcutaneous injectable anaesthesia. **B:** The syringe loaded with the therapeutic agent (for illustration purpose blue dye was used here). **C:** The injecting needle was loaded with cells (here loaded with the dye) and a mark was made at tip of the needle tip. **D:** The hair was shaved and the kidney was located using the fingers by palpating**. E:** After locating the kidney, the organ was held firmly to avoid slipping. **F:** The needle was inserted up to the mark on the needle and the cells were slowly injected into the kidney through the skin.

The animals were anesthetised with ketamine. Anaesthesia was confirmed by the lack of movement by pinching the tail or leg (Fig. 1A). Thereafter, the following procedure was performed on the animal (Fig. 1):

1 Syringe was loaded with the diluted drug or cells (for GFP tagged cells, 0.5 million cells were diluted in 50 μl PBS) (Fig. 1B).
2 A mark was made 3 mm from the tip of the needle to indicate the depth at which the needle will be inserted into the kidney (Fig. 1C).
3 The animal was placed on the heat pad.
4 Upon anaesthesia confirmation, the hair at the surgical site was removed using the electrical clippers or shavers (Fig. 1D).
5 The skin after removing the loose hair was cleaned by wiping with ethanol using gauze.
6 The kidney will be visible slightly after cleaning (for white skin animals like NOD/SCIDS)
7 Following that, the kidney was located using the fingers by palpating (Fig. 1D).
8 After locating the kidney, the organ was held firm to avoid slipping (Fig. 1E).
9 The needle was inserted into the kidney directly through the skin up to the mark on the needle (Fig. 1F).
10 The cells were slowly injected leaving a 2 s pause for every 10 μl injected.
11 If required, the procedure was repeated on the other side of the body to facilitate the injection into the other kidney.
12 The animals were observed for recovery.

### Post-injection care

The animals were returned to their cages and were monitored for their recovery. On monitoring the mice were immobile for 1 h (due to post-injection and anaesthetic recovery) and following that they appeared normal. Wet food and water were provided for 3 days on floor and the animals were observed for food and water intake, weight loss, mobility and general activity. After 3 days mice were treated normally (food and water on cage top).

### Tissue collection and analysis

After one week, the animals were humanely killed by a single overdose injection of pentobarbitone. The organs were perfused by PBS and fixed by 4% paraformaldehyde. Thin sections of 30–40 μm were made using cryostat and the cut sections were transferred to PBS solution or to antifreeze solution for long term storage. For analysis, random sections were washed in fresh PBS, mounted on glass slides and viewed under the fluorescent microscope. For H & E staining, sections were prepared automated and the image was taken using Nanozoomer S60 (Hamamatsu).

## Results and discussion

**Fig. 2:**
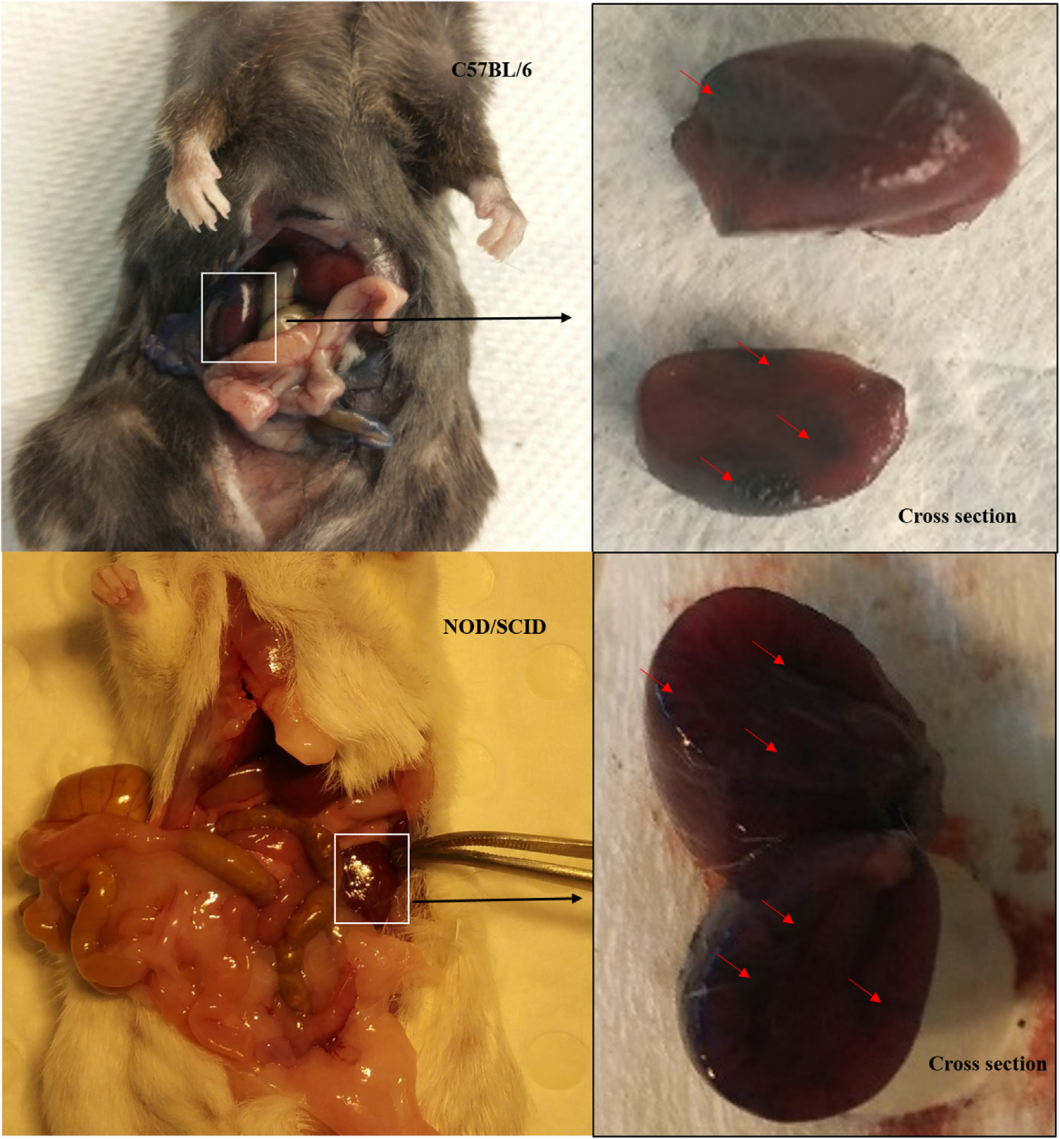
**Kidney analysis. A:** Kidney anatomy after post-mortem in C57 and NOD/SCID mice.

**Fig. 3:**
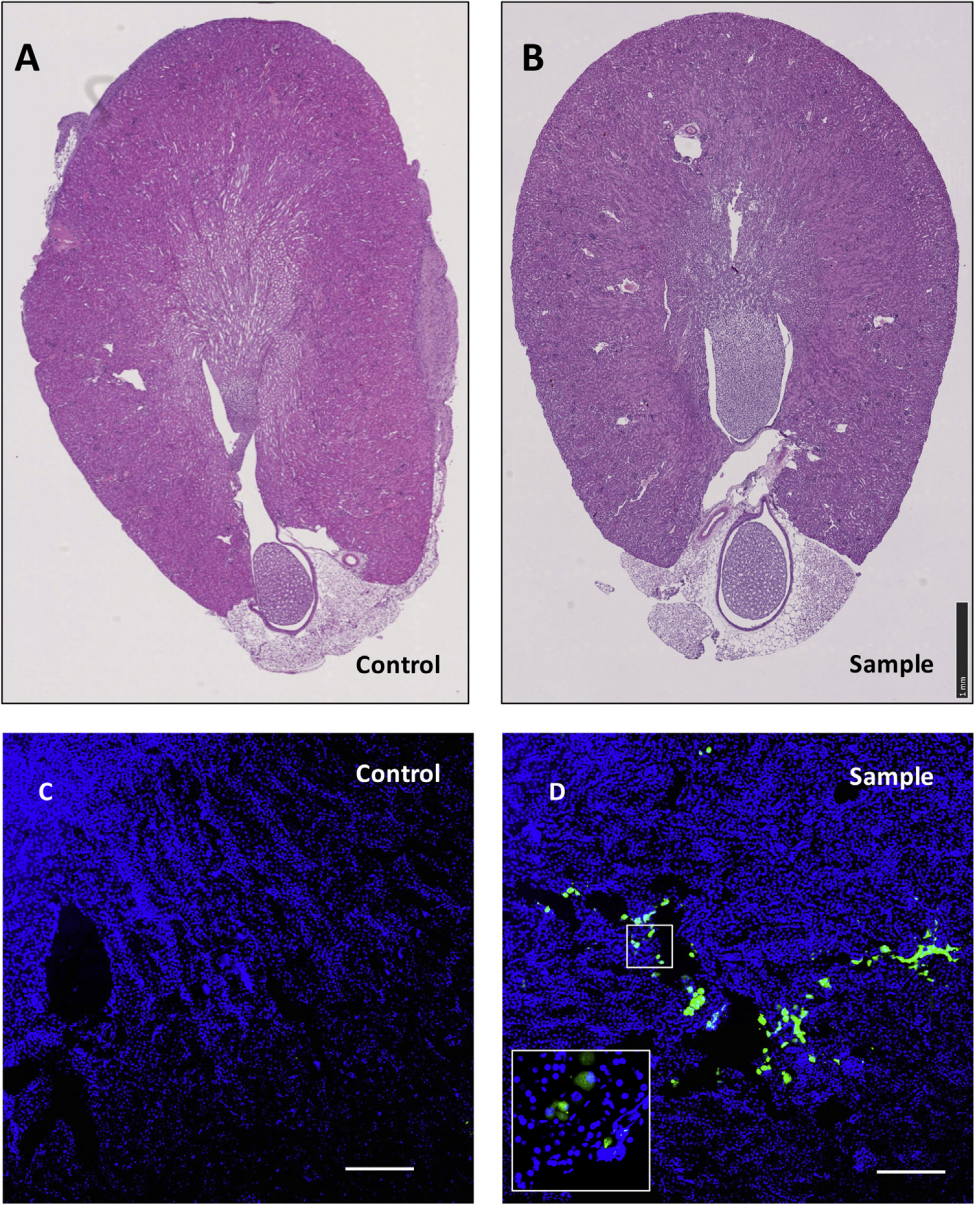
**Tissue Analysis:** A, B: H & E staining reveals normal structures in the control and sample kidney sections. C, D: histology analysis reveals the presence of GFP tagged cells by the method. Green colour indicates the tagged cells. Blue color indicates nuclear staining by DAPI. Scale bar: 100 μm.

We were able to perform the procedure on C57BL/6 and NOD/SCID mice successfully. The subjects were not given analgesics throughout the study and the welfare impact of the procedure was reduced to minimum. The animals appeared without complication 3 days after the injection. Mice kidney was injected with blue dye and with GFP-tagged human cells to compare the feasibility of the procedure for the delivery of drugs and stem cells respectively. On post-mortem, the presence of blue dye was investigated in the kidney after the injection (Fig. 2). Furthermore, stem cell injection and histological analysis demonstrated the presence of tagged cells (Fig. 3), suggesting the method can be used for engraftment studies in renal tissues.

The key step of the procedure is identifying and firm holding of the kidney. There is a high chance that the organ may slip while you try to identify and hold it for injection (Fig. 3). For a successful injection the kidney is needed to be held gently but firm enough to fix the position through the fingers. Kidney can be diagnosed through its solid and firm structure unlike the rest of soft organs inside the abdomen. Besides, for white haired animals, the organ can be viewed as dark colour outside the skin under a fibrotic light (Step 3 in the graphical abstract Fig). Therefore, the organ can be identified easily after palpitation to perform reliable injection. Nevertheless, care needs to be taken not to hold the organ firm for long time, to avoid damaging the tissue or causing inflammation. Besides, it is also equally important not to insert the needle far deep through the organ, which can result in the rupture of the blood vessels and inflammation. However, with steady hands the whole procedure can be performed within 15 min. Long-term analyses are required to see the survival of the animals through this procedure and the functionality of the injected agents.

## Conclusion

In this study, we have presented a new approach for the delivery of stem cells and drugs into the mice kidney. The significant highlight of the procedure is that the method does not incur any major physiological challenge for the animal (stiches, pain, infection and analgesics). The procedure is simple and can be carried out by one person in 15 min and without adverse events. Besides, the whole procedure distresses the animal significantly short time during the handling. Nevertheless, the method does not involve any surgery for the transplantation and therefore the animals’ survival rate is expected to be better than the surgical based procedure reported for targeted renal delivery. With further studies, the method may also be validated for the delivery of nanomaterials, metabolites and hormones; and will be advantageous in studies where more than one delivery is required. Nevertheless, detailed tissue analysis needs to be carried out to understand the survivability of the transplants by this method. Besides, the method is not recommended for procedures where the drugs need to be introduced to particular regions inside the kidney (for instance, to deliver inside the vascularised parenchyma). Though the study demonstrates the feasibility of the procedure in mice, the method can also be performed on rats.

## Funding

University of South Australia (UniSA), D&R Pharmaceutics China.

## Conflicts of interest

The authors indicate no conflict of interest
